# Quantifying and characterizing major DOC fractions in water treatment processes: A simplified SPE method without recovering sorbed compounds

**DOI:** 10.1002/wer.70047

**Published:** 2025-02-20

**Authors:** Saeideh Mirzaei, Beata Gorczyca

**Affiliations:** ^1^ Department of Civil Engineering University of Manitoba Winnipeg Manitoba Canada

**Keywords:** hydrophilic organic matter, molecular weight distribution, specific UV_254_ absorbance, trihalomethane formation potential, water‐dissolved organic carbon

## Abstract

Practitioners often use specific UV_254_ absorption (SUVA) as an indicator of hydrophobic (HPO) dissolved organic carbon (DOC) to evaluate removal efficiency and estimate trihalomethane formation potential (THMFP). However, current fractionation methods, such as solid‐phase extraction (SPE), primarily focus on recovering sorbed compounds, and assume that treatment impacts only the quantity, not the characteristics, of DOC fractions. Additionally, varying recovery rates and inconsistent fractionation pH definitions complicate cross‐study comparisons of hydrophilic and HPO DOC composition. To address these issues, we tested three pH fractionation approaches (pH 3, pH 7, and sequential adjustment) and observed significant differences in DOC content, SUVA, and specific THMFP (STHMFP) between SPE filtrates at pH 3 and pH 7, which were most likely because of ionizable acidic DOC compounds becoming HPO at lower pH levels.

Based on these findings, we developed a new fractionation method to estimate the quantity and characteristics of major DOC fractions—hydrophilic neutral (HPIN), total acidic (TA = HPOA + HPIA), and hydrophobic neutral (HPON)—without the need to recover sorbed fractions. Applying this method in a conventional coagulation/softening plant revealed HPON decreased while the relative amounts of HPI and TA increased after the treatment. However, the treated water HPI exhibited significantly higher STHMFP and contained approximately twice the proportion of low‐molecular‐weight compounds than raw water HPI, highlighting significant changes in both the content and properties of DOC fractions throughout the treatment process. Our study indicates that the contribution of HPI DOC fraction to SUVA and STHMFP in treated water is greater than that of HPO DOC.

**Practitioner Points:**

- A two‐stage ENV to estimate major DOC fractions without recovering sorbed compounds.
- One ENV cartridge at pH 3 can effectively isolate HPI DOC, replacing sequential ENV.
- Coagulation and lime/soda softening altered characteristics of DOC fractions.
- HPI DOC in treated water contributes to SUVA and STHMFP more than HPO fraction.

## Graphical abstract

Assessing the quantity and characteristics of HPI DOC in Bond Elut ENV filtrates at pH 3 and 7.

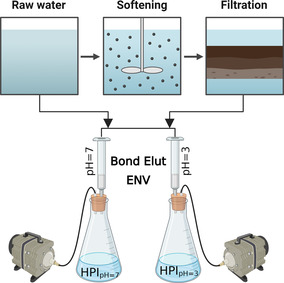

## INTRODUCTION

Natural organic matter (NOM) is a complex mixture of organic materials present in water, typically classified into particulate and dissolved organic carbon (DOC) based on its retention or passage through a 0.45‐μm filter. DOC in water poses several challenges for drinking water providers, including membrane fouling (Azzeh, 2014; Kwon et al., 2005), increased demand for chemicals such as coagulants (Sillanpää et al., 2018), and the need for more frequent use of membrane clean‐in‐place (CIP) agents (Alresheedi et al., 2019; Gruskevica & Mezule, 2021; Kuzmenko et al., 2005). More importantly, DOC can lead to the formation of potentially carcinogenic and regulated disinfection by‐products (DBPs) such as trihalomethanes (THMs) generating potentially carcinogenic and regulated disinfection byproducts (DBPs)‐trihalomethanes (THMs) (Richardson, 2003; Wang et al., 2014). As a result, a major focus of drinking water research has been the detailed characterization of DOC to better understand its behavior during treatment processes.

The characterization of DOC by practitioners often relies on tools like specific UV_254_ absorption (SUVA) per milligram of carbon (SUVA = UV_254_ × 100/DOC). SUVA has been extensively employed to estimate the hydrophobic (HPO) and high‐molecular‐weight fractions of NOM and served as an important indicator for the treatability of NOM in various processes, including (enhanced) coagulation (see Table S1) (Archer & Singer, 2006; Crittenden et al., 2012; Edzwald & Tobiason, 1999). Moreover, SUVA is recognized as a valuable parameter for assessing the aromatic content of DOC, as it is strongly correlated with the relative abundance of aromatic and highly conjugated organic compounds (Weishaar et al., 2003). Beyond SUVA, fractionating DOC into HPO and hydrophilic (HPI) components provides practitioners with numerous benefits.

Isolating HPO and HPI DOC fractions facilitates comparisons of DOC across different water sources (Fabris et al., 2008; Finkbeiner et al., 2020; Goss et al., 2017; Kent et al., 2014), enables the assessment of DOC removal during treatment processes, and aids in analyzing biodegradability of DOC fractions following advanced oxidation processes (Phungsai et al., 2021; Qu, Liang, He, et al., 2012; Ratpukdi et al., 2009; Zhang et al., 2012). Moreover, it helps identify components responsible for membrane fouling and the formation of disinfection byproducts (DBPs) (Hua & Reckhow, 2007; Kwon et al., 2005; Phattarapattamawong et al., 2016; Yamamura et al., 2014).

Several techniques have been developed to isolate HPO and HPI fractions of DOC. Early methods relied on bulk resins, but later advancements introduced the use of pre‐packed solid‐phase extraction (SPE) cartridges. Leenheer (1981), pioneered a method where a filtered water sample at pH 7 was passed through an Amberlite XAD‐8 resin column to retain the hydrophobic neutral (HPON) fraction. The effluent was then acidified to pH 2 and recirculated through the same column to capture the hydrophobic base (HPOB) and hydrophobic acid (HPOA) fractions. Subsequent processing of the effluent through cation‐ and anion‐exchange resins isolated the hydrophilic base (HPIB) and hydrophilic acid (HPIA) fractions, respectively, while the hydrophilic neutral (HPIN) fraction remained unretained by any resin. In a similar approach, Bose and Reckhow (1998) used the XAD‐8 column to retain all HPO DOC without pH adjustment, later eluting the HPON, HPOA, and HPOB fractions using methanol, NaOH, and HCl, respectively.

J.‐P. Croué (2004) Croué further refined this method by first separating the DOC into colloidal and non‐colloidal components using a 3.5‐kDa dialysis membrane, and then dividing them into HPO, transphilic (TP), and HPI fractions using XAD‐8/4 resins at pH 2. Later, bulk resins were replaced by SPE cartridges to reduce the labor‐intensive preparation and cleaning of bulk resins, especially for smaller water volumes. In many studies, a single SPE cartridge at pH 2–3 was used to isolate HPO and HPI DOC (Dittmar et al., 2008; Goss, 2019; Kaiser et al., 2003; S. Kim et al., 2003; Leenheer et al., 2000; Phungsai et al., 2021; Schwede‐Thomas et al., 2005). Ratpukdi et al. (2009) combined three HPO ENV cartridges with sequential pH adjustments to 7, 2, and 10, assuming the effluent to be HPI DOC, which was further divided into HPIB, HPIA, and HPIN fractions using StrataX‐C and StrataX‐AW SPE cartridges. The different methods applied for isolation of HPO and HPI DOC make it difficult to compare the results reported in the literature.

**FIGURE 1:**
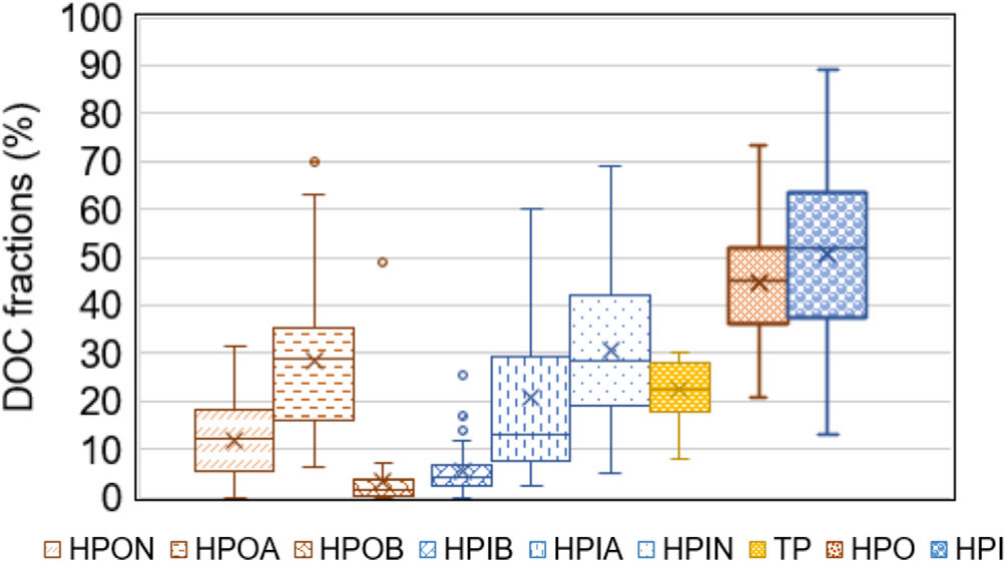
Box and whisker plot illustrating the content of DOC fractions isolated using bulk resins and SPE cartridges from freshwater sources and various stages of water treatment plants (WTPs), as summarized in Table S2. The crimson red bars represent hydrophobic organic carbon (HPO) fractions, while the blue bars correspond to hydrophilic organic carbon (HPI) fractions. The number of data points for each fraction is as follows: 49 for HPON, HPOA, HPOB, and HPIB; 50 for HPIA and HPIN; 14 for the transphilic (TP) fraction; and 83 for the HPO and HPI. DOC, dissolved organic carbon.

We reviewed studies that isolated DOC fractions from freshwater sources and various stages of water treatment (summarized in Table S2 and illustrated in Figure 1). The review revealed that, across both raw and treated samples, HPI DOC generally comprises a slightly larger proportion of total DOC, with a mean value of 51%, compared to 45% for HPO DOC. Among individual fractions, HPIN accounts for the largest share, with a mean of 30%, followed by HPOA as the second‐largest fraction, with a mean of 28%. HPIA ranks third, with a median of 13% and a mean of 21%, exhibiting considerable variability across studies. HPON contributes a median and mean of 12%, indicating that while neutral HPO compounds are significant, they are less abundant than HPI components. HPIB and HPOB are the smallest contributors, with median values of 4% and 2%, respectively. Overall, the major DOC fractions identified across studies are HPIN, total acidic components (HPOA and HPIA), and HPON, which together account for the bulk of the DOC composition in water sources.

The contribution of HPI DOC is particularly noteworthy in both raw and treated water. In some studies, the proportion of HPI DOC in raw water exceeded 70%, highlighting the dominance of HPI components in certain freshwater sources (Goss & Gorczyca, 2013; Kent et al., 2014). After treatment, the HPI fraction often remains substantial, with some studies reporting that it constitutes up to 87% of the finished water DOC (Marhaba et al., 2000). The HPI fractions are generally more resistant to removal by conventional treatment methods compared to HPO fractions (Finkbeiner et al., 2020; H. C. Kim & Yu, 2005; Kitis et al., 2002). The HPO and HPI nature of DOC also influences the type and reversibility of ultrafiltration (UF) membrane fouling (Qu, Liang, Wang, et al., 2012), with HPI fractions identified as key contributors to irreversible fouling (Yamamura et al., 2014). While HPO compounds have been shown to be relatively stable across seasons, HPI content is more susceptible to seasonal variations, adding uncertainty to the treatment process (Phattarapattamawong et al., 2016) and fluctuations in water quality during climate change events (Blackburn et al., 2023). Therefore, understanding the characteristics of the HPI fractions is essential for optimizing treatment processes and mitigating operational challenges.

Several studies have indicated elevated trihalomethane formation potential (THMFP) levels for HPO fractions and identified a positive correlation between DBP formation potential and hydrophobicity (Finkbeiner et al., 2020). However, significant THMFP has also been noted for HPI DOC compared to HPO fractions (J.‐P. P. Croué et al., 2000; W. J. Huang & Yeh, 1997; Kitis et al., 2002; Matilainen et al., 2011). Moreover, while some researchers discovered that low molecular weight (MW) compounds (<1 kDa) exhibited higher reactivity with chlorine (Liu et al., 2022; Özdemr, 2014), others identified that the variations in THMFP among coagulated waters correlated with the concentrations of high MW compounds (Fabris et al., 2008; H. C. Kim & Yu, 2005) (Kitis et al., 2002). While biopolymers (BP) (MW > 10 kDa), typically categorized as the HPI fraction of DOC and containing polysaccharides and aminosugars, are known for their low UV absorbance (Kent et al., 2014; Kwon et al., 2005), both BP and UV_254_ were found to have strong correlations with the membrane fouling index (W. Huang et al., 2019).

These variations in the characteristics of HPI DOC across studies could stem from inconsistencies in defining HPI DOC. For instance, Bose and Reckhow (1998) classified the effluent from a HPO resin at pH 7 as HPI DOC, while the sequential‐SPE method identifies the effluent from three ENV cartridges, with intermittent pH adjustments, as HPI DOC. In contrast, the single SPE/resin method considers the SPE eluate at pH 2–3 as the HPI fraction. Because the hydrophobicity of natural water DOC is strongly affected by pH because of the many ionizable and interacting functional groups within its structure (Chen et al., 2015; Newcombe & Dixon, 2006; Patriarca et al., 2020; Rosario‐Ortiz et al., 2007), differences in fractionation pH can significantly affect the estimation of both the quantity and characteristics of the isolated HPI fractions.

Moreover, the focus on the HPO fraction in many studies—especially those using single resin or SPE at pH 2–3—has resulted in the HPI fraction being discarded in the SPE filtrate and overlooked (Dittmar et al., 2008; Grasset et al., 2023; Petras et al., 2017; Phungsai et al., 2021). The HPI DOC fraction is predominating in the effluents of the conventionally treated waters, therefore contains majority of DBP precursors. In addition, the main drawback of the reviewed methods is that they require eluting the DOC fractions from the resin, which complicates cross‐study comparisons because of varying recovery rates. In some cases, DOC deficits or surpluses have ranged from 30% to 13%, respectively (Marhaba et al., 2000; Yamamura et al., 2014). Furthermore, eluting fractions rather than using the resin filtrate can introduce inconsistencies in the characteristics of DOC fractions, as eluents or resins may selectively target specific compounds and interact differently with various DOC components (Aiken et al., 1992; Nebbioso & Piccolo, 2013). Therefore, we identified the need to focus on the HPI fraction in SPE effluents (rather than HPO fraction adsorbed) and investigate the effects of fractionation pH on the characteristics of isolated DOC fractions.

### Objectives

The objective of this study was to evaluate and compare the DOC fractionation methods and results reported for water treatment systems. We applied three fractionation methodologies using ENV cartridges at pH 7, pH 3, and sequential pH adjustments. Fractionation and analyses were conducted on both raw and treated water—following coagulation, softening, and sand filtration—to assess changes in HPI concentration, SUVA, MW distribution, and specific trihalomethane formation potential (STHMFP) induced by conventional water treatment processes. We also proposed a fractionation method that estimates the quantity and characteristics of major DOC fractions, including HPIN, total acidic (TA = HPOA + HPIA), and HPON, without the need to recover the sorbed fractions.

## MATERIALS AND METHODS

### Sampling protocol

Three sets of water samples were obtained from a conventional lime/soda softening water treatment plant (WTP) in Brandon, Manitoba, Canada, on February 2, 15, and March 1, 2022. This 1‐month sampling strategy helped minimize seasonal water quality fluctuations while enabling some preliminary analyses. The plant is supplied by the Assiniboine River and the treatment process involves inline coagulation with alum, followed by lime/soda softening clarifiers, recarbonation, and single‐media silica sand filtration. Sampling was conducted at three points: raw water intake (Raw), after the softening clarifier (Soft), and before chlorination (Soft/Filt) (Figure S1).

### SPE fractionation methods

**FIGURE 2:**
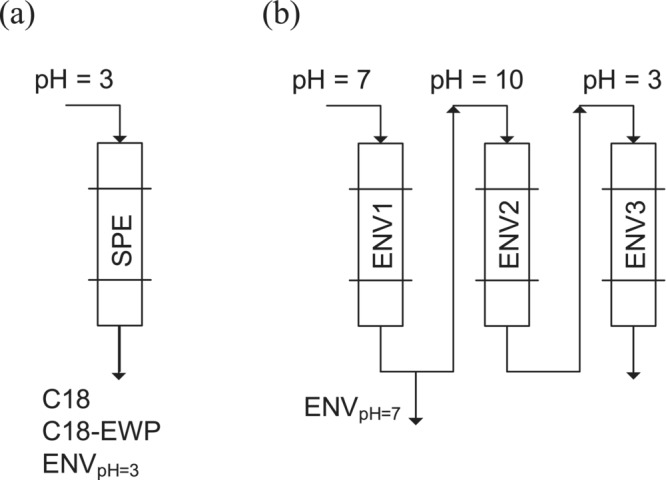
Fractionation approaches to isolate HPI DOC: (a) single‐SPE at pH = 3 and (b) single‐ENV at pH = 7 and sequential‐ENV. HPI DOC, hydrophilic dissolved organic carbon.

In this study, three fractionation pH approaches were implemented using Bond Elut ENV SPE cartridges (Figure 2): pH = 3, pH = 7 (corresponding to the initial step in the Ratpukdi et al., 2009 method and equivalent to HPI isolated by Bose and Reckhow [1998]), and sequential pH adjustment (similar to the first three steps in the Ratpukdi et al. [2009]). Bond Elut ENV was the primary SPE type used across these pH approaches, as it was effectively employed by Ratpukdi et al. (2009) to separate HPO from HPI DOC (Ratpukdi et al., 2009). In addition, C18 and C18‐EWP SPE cartridges were compared with ENV at pH = 3. This comparison was motivated by previous studies demonstrating that C18 retains a comparable amount of HPO organic carbon to XAD resins utilized by Bose and Reckhow (1998), Goss (2019), and Schwede‐Thomas et al. (2005). However, the pore size of C18 SPE is smaller than ENV. Therefore, we included C18‐EWP with the same resin type as C18 but with an extra wide pore size of 500 Å, close to ENV's with 450 Å (refer to Table S3). We decided not to utilize Bond Elut PPL, another frequently employed SPE cartridge in water DOC fractionation (Goss et al., 2017; Phungsai et al., 2021) because its modified surface would retain concurrently highly and poorly ionizable materials, which would not allow us to investigate the effect of pH on ionizable HPO DOC (Patriarca et al., 2020).

Humic acid sodium salt (Aldrich, CAS#68131‐04‐4) has been used as a standard. Aquatic humic acid was used previously (Bratskaya et al., 2004; Furukawa et al., 2014; Imyim & Prapalimrungsi, 2010; Pham et al., 2020; Tao et al., 2011). It serves as a fundamental constituent of humic substances, characterized by a well‐defined structure and chemical formula. Utilizing complex standards like NOM, HA, and FA IHSS may not have enabled us to distinguish between the behavior exhibited by natural water DOC and that of a more simplified standard component. The cartridges' influent and effluent were analyzed for DOC, UV_254_, THMFP, and MW distribution using high‐performance size exclusion chromatography (HPSEC), with the pH adjusted at 7 to counterbalance the pH interference with these analyses. We did not elute any HPO from the cartridges, and HPO DOC was calculated by deducting the HPI DOC in the SPE effluent from the total DOC concentration.

### Applied analytical techniques

#### DOC and UV_254_ measurements

DOC was quantified using a Teledyne Tekmar Fusion Total Organic Carbon analyzer (Mason, Ohio, USA). UV measurements were conducted using a UV/visible spectrophotometer, Ultraspec 2100 Pro (GBC Scientific Equipment, Australia), equipped with a 1‐cm quartz cell. All UV measurements were performed on samples filtered through a pre‐washed 0.45 μm polyethersulfone (PES) filter, with pH adjusted to 7 to mitigate the influence of pH and particles on UV absorbance.

#### Determining THMFP

For THMFP determination, samples filtered through a 0.45 μm filter were adjusted to a pH of 7 and subjected to super‐chlorination with 30 mg/l of free chlorine. This process was conducted in 70‐ml amber bottles with no headspace, and the samples were incubated for 7 days at room temperature (21°C). The chlorine dose was selected to ensure adequate chlorine residuals in 7 days (even in raw water) according to the chlorine demand of similar source water (Goss et al., 2017; Mirzaei & Gorczyca, 2020). The chlorine residual was quenched by sodium thiosulfate. Four THMs were quantified, namely chloroform (CF), bromodichloromethane (BDCM), dibromochloromethane (DBCM), and bromoform (BF), with their combined total considered as the sum of THMs.

THMs were extracted with pentane following the standard method 6232B (APHA, 2012) and analyzed using an Agilent 7890A Gas Chromatography (GC) system (Agilent Technologies, Santa Clara, California). The GC was equipped with a CombiPAL CTC Analytics autosampler, an electron capture detection (ECD) and a DB‐5 Agilent column (P/N 123‐5033). The injection temperature was set to 220°C with a split ratio of 25, and an injection volume of 1 μl was used. The ECD detector temperature was held constant at 220°C. The combined flow rate of the carrier gases (helium and nitrogen) passing through the ECD was set to 60 ml/min.

#### HPSEC MW distribution analysis

The HPSEC analysis was performed using a Waters e2696 HPLC module connected to a Waters 2998 photodiode array (PDA) detector and an s2000 column (7.8 × 300 mm) from the BioSep‐SEC series capable of separating 1–300 kDa. The sample injection volume was 30 μl, and a mobile phase consisting of sodium phosphate buffer (25 mM) was used at a flow rate of 1 ml/min. Standards of polystyrene sulfonated salts (PSS) with MWs of 1.7, 5.0, 7.5, and 16 kDa (Scientific Polymer Products Inc., Ontario, NY) were prepared at a concentration of 1 g/l in deionized water and used to convert retention time in minutes to apparent molecular weight (AMW) in kDa.

We employed a normalized HPSEC (NHPSEC) approach, wherein the HPSEC UV intensities were divided by the nanograms of the injected DOC (injection volume × DOC concentration). This normalization method mitigated the influence of sample DOC concentrations on the chromatogram intensity. The molecular weight distribution within each sample was assessed by dividing the integrated area of each region by the total integrated area of the chromatogram. This analysis was conducted using MATLAB R2021a (MathWorks, Natick, MA, USA). The HPSEC regions included low‐molecular weight compounds and building blocks (LMWs and BB) ranging from 0.1 to 1 kDa, humic substances (HS) ranging from 1 to 10 kDa, and biopolymers (BP) greater than 10 kDa, as defined by Brezinski and Gorczyca (2019).

#### Statistical analysis

Statistical significance was assessed using single‐ and two‐factor analysis of variance (ANOVA), with or without replication, to compare the effects of different factors on DOC fractions. For example, two‐factor ANOVA was used to compare HPI DOC concentrations across three different resins (rows) and between raw and treated (Soft/Filt) water (columns), providing six data points in total. The mean values were compared in these analyses, as ANOVA assumes normally distributed data and homogeneity of variances. The assumptions of normality and equal variances were tested using the Shapiro–Wilk test and Levene's Test performed in Excel using the Real Statistics add‐in. The Excel Data Analysis Tool was then used for the statistical calculations, with *p*‐values below 0.05 considered statistically significant.

## RESULTS AND DISCUSSION

### WTP baseline data

General water‐quality parameters are presented in Table S4. The initial DOC concentration in the raw water was found to be 7.74 ± 0.40 mg/l. Coagulation, softening, and sand filtration processes reduced DOC by 42 ± 5%, resulting in a Soft/Filt DOC concentration of 4.5 ± 0.6 mg/l. The observed DOC removal fell within the range reported by other studies (Bose & Reckhow, 2007; Hussain et al., 2013; Sillanpää et al., 2018). The UV_254_ of raw water was 0.213 ± 0.01 cm^−1^ and sharply decreased by 64 ± 4%, reaching a value of 0.079 ± 0.008 cm^−1^ after the coagulation/softening. Because sand filtration did not have a significant effect on DOC and UV_254_, as expected, we focused on the Soft/Filt sample as the final water before chlorination rather than Soft water in the following discussions.

### Effect of resin type and pore size on the SPE filtrate at pH = 3

The HPI fractions from the Raw, Soft/Filtered, and HA standard samples were isolated at pH 3 using three SPE cartridges: ENV, C18 EWP, and C18 (refer to Table 1). Our analysis showed no significant differences in the quantity (*p* = 0.97) and characteristics (*p* > 0.05) of the isolated HPI fractions across the different SPE resin types and pore sizes. Consequently, the retention of HPO DOC by both silica‐ and polymer‐based HPO resins was most likely a result of HPO interactions with the DOC molecules rather than size exclusion. The HPI content of the HA standard was also not affected by the type and pore size of the SPE resin and was measured at 73.2 ± 1.0%. Therefore, we focused on the results obtained using the ENV cartridge from this point onward.

**TABLE 1:** DOC, UV_254_, and SUVA of HPI fractions isolated at pH = 3 using three different SPE cartridges.

Sample	SPE type	HPI DOC (mg/l)	HPO DOC (mg/l)	UV_254_ (cm^−1^)	SUVA l/(mg‐C·m)	% HPI
**Raw**	**None**	**7.74 ± 0.40**		**0.213 ± 0.010**	**2.76 ± 0.24**	
	ENV	1.20 ± 0.28	6.75 ± 0.11	0.166 ± 0.041	13.8 ± 0.21	15.0 ± 3.18
	C18‐EWP	1.35	6.48	0.208	15.4	17.5
	C18	1.28	6.55	0.195	15.2	16.4
**Soft/Filt**	**Unfractionated**	**4.59 ± 0.41**		**0.079 ± 0.008**	**1.67 ± 0.08**	
	ENV^a^	1.21 ± 0.11	3.74 ± 0.08	0.081 ± 0.002	6.67 ± 0.44	24.4 ± 2.13
	C18‐EWP	0.87	4.09	0.083	8.31	17.5
	C18	0.86	4.10	0.072	9.65	17.3
**HA Std.**	**Unfractionated**	**3.86 ± 0.029**		**0.487 ± 0.001**	**12.63 ± 0.133**	
	ENV	2.86	1.00	0.448	15.6	74.3
	C18‐EWP	2.79	1.07	0.434	15.5	72.4
	C18	2.81	1.05	0.442	15.7	73.0

*Note*: The first row in bold for each section represents the data for unfractionated samples. The ± symbol indicates the standard deviation from analysis and fractionation conducted on three sampling dates (February 2, 15, and March 1). HPI DOC, hydrophilic dissolved organic carbon; HPO DOC, hydrophobic dissolved organic carbon; SUVA, specific UV_254_absorption.

^a^ It is the average of the ENV_pH = 3_filtrate of Soft and Soft/Filt samples.

The DOC concentration in the ENV filtrate at pH = 3 was statistically similar for both raw and Soft/Filt water, measuring 1.2 ± 0.28 and 1.21 ± 0.11 mg/l, respectively. In contrast, the concentration of sorbed DOC on the resin decreased significantly from 6.75 ± 0.11 mg/l in raw water to 3.74 ± 0.08 mg/l after coagulation, softening, and filtration. The reduction in the content of HPO DOC during coagulation and softening has often been attributed to the preferential removal of HPO DOC (Fabris et al., 2008; Sillanpää et al., 2018). However, despite the similar HPI content in both Raw and Soft/Filt water (~1.2 mg/l), their UV_254_ absorbance and SUVA values differed significantly. The raw water HPI had a UV_254_ of 0.166 ± 0.041 cm^−1^ and a SUVA of 13.8 ± 0.21 l/mg‐C·m, while the Soft/Filt HPI showed a lower UV_254_ of 0.081 ± 0.002 cm^−1^ and a SUVA of 6.67 ± 0.44 l/mg‐C·m. This reduction in both UV_254_ and SUVA suggests that the treated HPI DOC underwent structural changes, resulting in decreased aromaticity and fewer UV‐active components in the HPI fraction.

The comparison between HPI fractions and unfractionated samples revealed significant changes induced by fractionation. The HPI DOC exhibited significantly higher SUVA values—up to six times greater than those of the unfractionated samples in both Raw and Soft/Filt water. This increase in SUVA post‐fractionation was mainly because of minimal changes in UV_254_ absorbance (SUVA numerator), combined with a substantial reduction in DOC concentrations (SUVA denominator). We confirmed that the elevated SUVA values in the HPI fractions were not because of background UV levels, as acidified DI water passed through prewashed SPE cartridges showed UV readings of <0.003 cm^−1^. Additionally, concentrations of UV‐absorbing inorganic components, like NO₃^−^ and iron, were negligible (Birkmann et al., 2018; Weishaar et al., 2003).

Similar elevated SUVA values, exceeding 10 l/(mg‐C·m), have been reported for the HPON (Kent et al., 2014) and the HPO basic DOC (Korshin, Benjamin, & Sletten, 1997) fractions, which were attributed to low DOC concentrations of <0.2 and ~0.02 mg/l, respectively. In the current study, the HA standard exhibited a substantial UV absorption of 0.487 ± 0.001 cm^−1^, leading to a high SUVA value of 12.63 ± 0.133 l/mg‐C·m at the DOC level of 3.86 mg/l. The UV absorbance of DOC compounds is attributed to intramolecular charge–transfer interactions within the DOC structure rather than the presence of independent chromophores (Del Vecchio & Blough, 2004; Korshin, Li, & Benjamin, 1997; Walpen et al., 2018). These interactions contributed to the complex UV absorbance behavior observed in total and fractionated natural water DOC samples as further discussed in Section 3.6.

### Effect of fractionation pH on the ENV filtrate

Table 2 compares the quantity, UV_254_, and SUVA of DOC in the effluent of the ENV cartridges using three pH approaches: pH 7, pH 3, and sequential pH adjustment. The results indicate that the sequential ENV approach produced statistically identical outcomes to those of the ENV HPI at pH 3 for both Raw and Soft/Filt samples. This suggests that the intrinsic HPI DOC, which remains HPI even at low pH, can be effectively isolated using a single ENV cartridge, eliminating the need for three consecutive cartridges. The ENV filtrates at pH = 7 in Raw and Soft/Filt samples were similar and measured at 3.76 mg/l, considering the differences between the DOC of unfractionated samples and the HPON in Raw and Soft/Filt were found to be 3.98 and 0.84 mg/l.

**TABLE 2:** HPI fractions of Raw and Soft/Filt using the ENV cartridge at different pH approaches.

Sample	SPE type	HPI DOC mg/L	HPO DOC mg/L	UV_254_ cm^−1^	SUVA L/(mg‐C·m)	% HPI
**Raw**	**Unfractionated**	**7.74 ± 0.40**		**0.213 ± 0.010**	**2.76 ± 0.24**	
	ENV _pH = 7_	3.76	3.98	0.207	5.50	46.6
	Sequential‐ENV	1.01	6.73	0.179	17.7	12.5
	ENV_pH = 3_	1.20 ± 0.28	6.54	0.166 ± 0.041	13.8 ± 0.21	15.0 ± 3.18
**Soft/Filt**	**Unfractionated**	**4.59 ± 0.41**		**0.079 ± 0.008**	**1.67 ± 0.08**	
	ENV_pH = 7_	3.76 ± 0.52	0.84 ± 0.01	0.075 ± 0.005	1.99 ± 0.14	81.6 ± 2.2
	Sequential‐ENV^a^	1.10	3.49	0.068	6.20	25.9
	ENV_pH = 3_	1.21 ± 0.11	3.74 ± 0.08	0.081 ± 0.002	6.67 ± 0.44	24.4 ± 2.13

*Note*: The unfractionated and ENV_pH = 3_ are repeated from Table 1 for a more convenient comparison. DOC, dissolved organic carbon; HPI hydrophilic; HPO, hydrophobic; SUVA, specific UV_254_ absorption.

^a^ The Soft/Filt water sample collected in March was not included because of low DOC of HPI left after three ENV cartridges: DOC = 0.29 mg/l, UV_254_ = 0.049 cm^−1^, SUVA = 18.7 l/(mg‐C·m), and % HPI = 5.8. The Soft water sample collected on March 1 was also fractionated to validate the data, and similar results were achieved: DOC = 0.36 mg/l, UV_254_ = 0.067 cm^−1^, SUVA = 18.7 L/(mg‐C·m), and % HPI = 7.27.

Also, at the pH of 7, the ENV effluent of the Raw water exhibited SUVA values that were 50% higher than the unfractionated sample, measuring 5.5 vs. 2.76 L/(mg‐C·m). However, the SUVA of Soft/Filt water did not change significantly upon fractionation by ENV_pH = 7_, measuring 1.99 vs. 1.67 L/(mg‐C·m). The differing impact of fractionation at pH = 7 on the SUVA of the Raw water compared to the Soft/Filt sample indirectly indicated that the sorbed DOC fractions by ENV cartridge of the Raw water had higher UV_254_ absorbability than those in the Soft/Filt sample, as discussed below.

### MW distribution of unfractionated samples and DOC fractions

**FIGURE 3:**
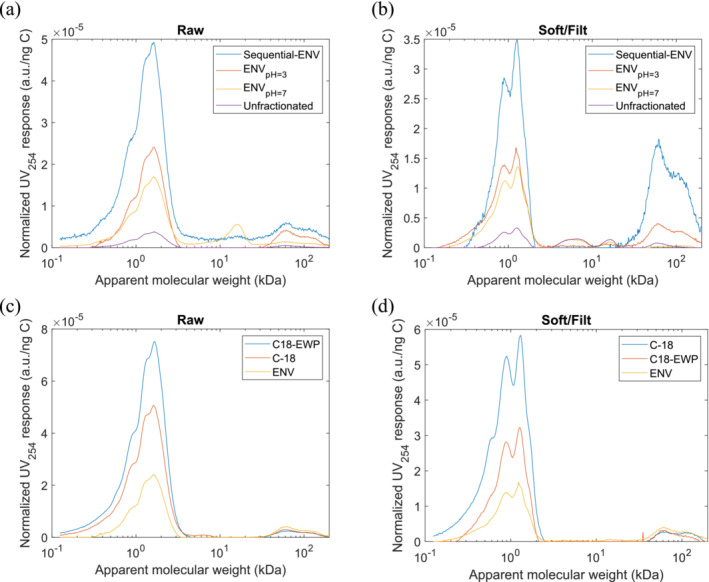
NHPSEC unfractionated and HPI fractions at varying pH approaches for (a) Raw and (b) Soft/Filt. The second row is HPI_pH = 3_ using different SPE cartridges for (c) raw and (d) Soft/Filt samples. The order of color legends matches the order of the chromatograms' peak at 1 kDa. HPI, hydrophilic; NHPSEC, normalized high‐performance size exclusion chromatography; SPE, solid‐phase extraction.

The NHPSEC chromatograms and MW distribution of total and SPE filtrates of Raw and Soft/Filt samples are provided in Figure 3. In the Raw NHPSEC chromatogram, two shoulders and one peak were observed around 1 kDa, followed by another distinct peak in the biopolymers region at 60 kDa. According to the MW distribution, the raw water consisted of 23.2% LMWs and BB, 67.3% HS, and 9.7% BP. The NHPSEC chromatogram of Soft/Filt water exhibited five distinct peaks, indicating a higher diversity of MW groups within the DOC compared to the raw sample. The process of coagulation and softening resulted in a modification of the MW distribution, primarily within the range of 2 to 20 kDa, and hence, significantly reduced the percentage of HS to 55.3% in the Coag/Soft sample.

All the HPI fractions of Raw and Soft/Filt waters had significantly higher peak intensities between 0.5 and 5 kDa than unfractionated samples that can be justified in two ways: (i) humic substances in HPI fractions had higher UV absorbance and (ii) HPI fractions had higher concentrations of HS and LMWs and BB between 0.5 and 5 kDa, which the former is more plausible and consistent with the higher SUVA of HPI fractions than unfractionated samples. Table 3 provides the MW distribution of Raw and Soft/Filt water HPI fractions calculated from the regional integration of chromatograms in Figure 3.

**TABLE 3:** The molecular weight distribution of Raw and Soft/Filt SPE filtrates.

Sample	SPE type	Injected DOC (ng)	LMWs and BB (%)	HS (%)	BP (%)
**Raw**	**Unfractionated**	**235**	**23**	**67**	**10**
	ENV_pH = 7_	113	22	58	21
	Sequential‐ENV	30	28	58	15
	ENV_pH = 3_	30	21	64	15
	C18‐EWP	41	30	67	3
	C18	38	30	65	5
**Soft/Filt**	**Unfractionated**	**149**	**24**	**55**	**21**
	ENV_pH = 7_	124	45	52	4
	Sequential‐ENV	33	28	32	40
	ENV_pH = 3_	9	42	36	22
	C18‐EWP	26	50	43	7
	C18	26	55	41	4

*Note*: BB, building block; DOC, dissolved organic carbon; HS, humic substances; LMW, low‐molecular weight; SPE, solid‐phase extraction.

Two‐factor ANOVA without replication revealed that the SPE filtrates of Soft/Filt water constituted significantly higher %LMWs and BB (*p* = 0.017) and lower %HS (*p* = 0.006) than that of Raw. Although %BP increased after coagulation and softening and upon some of the fractionation approaches, which was likely because of aggregation of some DOC compounds upon changing the pH, none of the differences between the %BP in unfractionated and HPI fractions of Raw and Soft/Filt were significant (*p* > 0.2). Also, the HPI fractions of the Soft/Filt sample exhibited significantly higher percentages of LMWs and BB and lower percentages of HS compared to the unfractionated Soft/Filt sample (*p* < 0.05). It suggests that the HPI DOC and the HPO fractions retained in the SPE cartridges likely had different MW distributions in the Soft/Filt sample, with the HPO fraction containing a more %HS and less %LMWs and BB compared to the HPI fraction. In contrast, fractionation did not alter the MW distribution of the Raw water sample, suggesting that the HPI and HPO fractions in the Raw water likely had similar MW sizes. Therefore, the data indicate that coagulation and softening induced distinct changes in the MW distribution of the DOC fractions.

### THM formation potential of unfractionated samples and DOC fractions

Itemized, total, and specific THMFP (STHMFP) of Raw and Soft/Filt water and SPE filtrates are provided in Table 4. The Soft/Filt water exhibited a lower THMFP compared to the raw water, measuring 474 ± 48 μg/l versus 366 ± 45 μg/l. However, it yielded a significantly higher STHMFP, with values of 77 ± 3 μg/mg‐C versus 60 ± 7 μg/mg‐C (one‐tailed *p* = 0.01 in a paired *t*‐test for means). Therefore, the lower THMFP of the Soft/Filt water sample is because of its lower DOC concentration not the lower reactivity of the organic compounds with chlorine. All the SPE filtrates had significantly higher STHMFP than the associated unfractionated samples. The Soft/Filt HPI (SPE filtrate at pH = 3) had statistically significant (*p* = 0.01) higher STHMFP than the HPI of Raw water. However, the ENV filtrate at pH = 7 of Soft/Filt water had lower STHMFP than that of raw water, 97 versus 153 μg/mg‐C. Therefore, the STHMFP of TA DOC probably decreased after coagulation/softening.

**TABLE 4:** THM formation potentials of unfractionated samples and HPI fractions isolated using various fractionation approaches.

Sample	SPE type	CF (μg/l)	BDCM (μg/l)	DBCM (μg/l)	THMFP^a^ (μg/l)	STHMFP (μg/mg‐C)
Raw	Unfractionated^b^	436 ± 40	35 ± 7	3 ± 1	474 ± 48	60 ± 7
	ENV_pH = 7_	520	51	5	576	153
	Sequential‐ENV	342	36	5	382	378
	ENV_pH = 3_	538	62	7	607	274
	C18‐EWP	526	51	5	582	431
	C18	444	45	5	494	385
Soft/Filt	Unfractionated^b^	331 ± 45	29 ± 3	7 ± 2	366 ± 45	77 ± 3
	ENV_pH = 7_ ^b^	331 ± 58	30 ± 6	5 ± 1	367 ± 64	97 ± 8
	Sequential‐ENV^c^	349	42	14	405	370
	ENV_pH = 3_	423	39	7	469	364
	C18‐EWP	404	41	8	454	523
	C18	389	39	7	436	506

*Note*: All samples underwent a single THMs measurement with duplicate THMs extractions, unless otherwise specified. BDCM, bromodichloromethane; CF, chloroform; DBCM, dibromochloromethane; THM, trihalomethane; THMFP, trihalomethane formation potential; SPE, solid‐phase extraction; STHMFP, specific trihalomethane formation potential.

^a^ The concentration of bromoform was below the detection limit in all samples.

^b^ The ± symbol indicates the standard deviation calculated from four replications—two sample sets and duplicate THMs extractions.

^c^ Sequential‐ENV of the March sample is excluded because of the low DOC concentration left following three ENV cartridges, 0.29 mg/l, resulting in STHMFP > 1000 μg/mg‐C.

It is worth noting that applied fractionation methods had a synergistic effect on STHMFP, that is, the contribution of STHMFP of SPE filtrates was higher than the STHMFP of the unfractionated sample ($x_{\mathrm{HPI}}\times S{\text{THMFP}}_{\mathrm{HPI}}>\text{STHMFP}$). Therefore, we could not conclude that the higher STHMFP of HPI fractions than unfractionated samples was because of the lower STHMFP of HPO fractions retained by SPEs. In contrast, we suspect that the reactivity of DOC components with chlorine may have increased upon fractionation because of fewer interactions with HPO compounds. Therefore, the STHMFP is likely not an intrinsic characteristic of the HPO or HPI DOC, similar to UV_254_ absorbance, and depends on the competing reactive sites in the sample.

### Correlations between UV‐DOC and SUVA‐STHMFP

**FIGURE 4:**
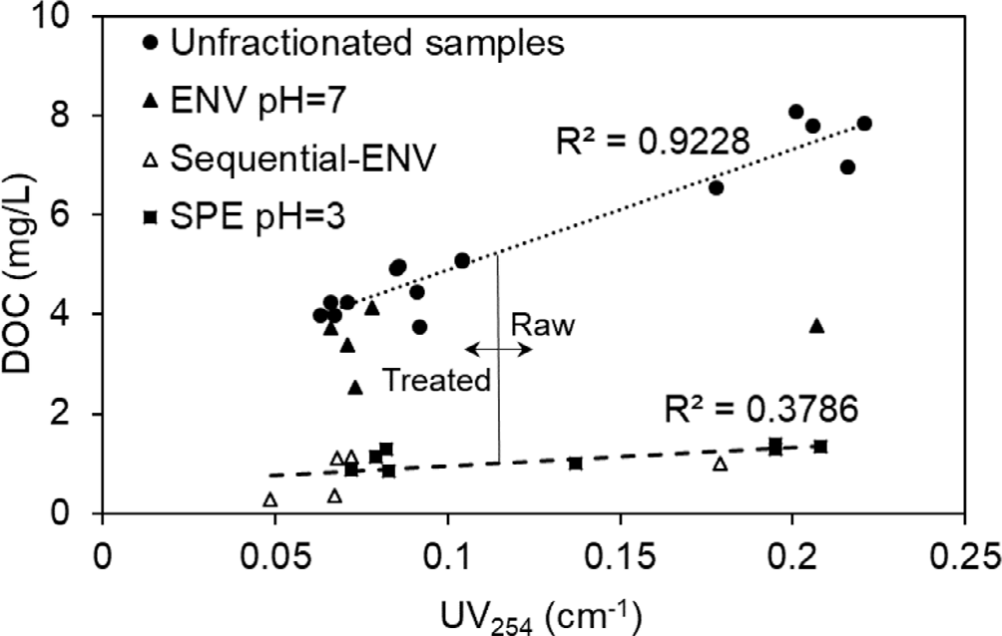
UV_254_ versus DOC for unfractionated samples and HPI fractions isolated by different fractionation approaches. All data for Raw, Soft, and Soft/Filt were included. The Soft and Soft/Filt samples are noted as “treated” in the figure's annotations. DOC, dissolved organic carbon; HPI, hydrophilic.

DOC versus UV_254_ of all unfractionated and SPE filtrates is shown in Figure 4. The data revealed a strong positive correlation between DOC and UV_254_ (*R* ^2^ = 0.92) for the unfractionated samples (Raw, Soft, and Soft/Filt) (solid circles in Figure 4). Unlike unfractionated samples, UV_254_ and DOC for SPE at pH = 3 and sequential‐ENV showed a poor correlation (*R* ^2^ = 0.38), and the data could not be correlated for ENV_pH = 7_ HPI (trendline plotted for solid triangles was not shown in Figure 4 because *R* ^2^ was 0.06). Contrary to traditional perceptions of humic substances as large macro polymers, some studies characterize them as intricate assemblies of relatively small, diverse molecules, each less than 1 kDa in size (Fiorentino et al., 2006). These humic molecules exhibit varying pK constants, influencing their protonation and hydrophobicity at different pH values (Angelico et al., 2014). Consequently, at a specific fractionation pH, the SPE isolation process may selectively retain certain humic compounds within the HPI fraction while excluding others. This selectivity led to a subset of compounds characterized by relatively consistent overall DOC concentrations but varying UV_254_ absorbance, and hence, poor correlation between DOC and UV.

In contrast to the natural water samples, humic acid sodium salt revealed a strong correlation (*R* ^2^ = 0.99) between UV_254_ and DOC, encompassing all data points for both unfractionated and single SPE fractionated samples (Figure S2). The discrepancy in the correlation patterns between DOC and UV observed in actual water samples versus the HA Std highlights the distinctive behaviors of natural organic matter and model compounds. The hydrophobicity of HA standard exhibits less dependency on pH variations, leading to a fractionation process that is less selective towards specific compounds. This reduced selectivity SPE isolation contributes to maintaining a high correlation between DOC and UV absorbance, as the isolation process does not significantly alter the composition of HA standard. The simplified structure and pH‐insensitive characteristics of HA standard offer valuable insights into the factors influencing the correlation between DOC and UV absorbance, such as molecular interactions.

**FIGURE 5:**
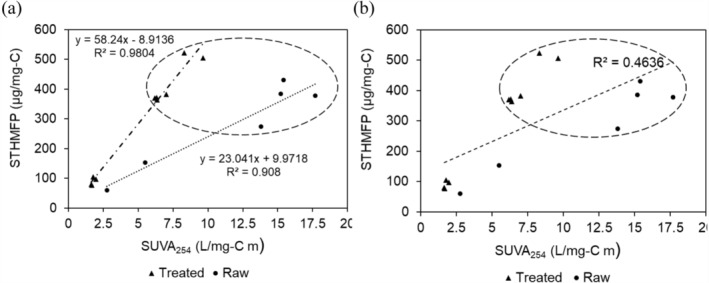
Correlation between SUVA and specific THM formation potential for unfractionated and SPE filtrates: (a) two separate trendlines represent Raw and Treated (Soft and Soft/Filt) water samples, and (b) a single trendline represents all data points. Data for HPI DOC isolated using SPE at pH 3 and sequential ENV are highlighted with a dashed circle. DOC, dissolved organic carbon; HPI, hydrophilic; SPE, solid‐phase extraction; SUVA, specific UV_254_ absorption; THM, trihalomethane.

The STHMFP of unfractionated raw, softened (Soft), and softened/filtered (Soft/Filt) water samples, along with their SPE filtrates, was plotted against SUVA (Figure 5). The correlation between SUVA and STHMFP varied depending on the type of water sample. When unfractionated samples and their SPE filtrates were analyzed separately for raw and treated (Soft and Soft/Filt) water (Figure 5a), SUVA exhibited a strong linear correlation with STHMFP, with *R* ^2^ values of 0.908 for raw water and 0.9804 for treated water. However, the slope of this relationship differed significantly between treated and raw samples. The trendline for the Soft/Filt samples had a much steeper slope (58) compared to the raw samples (23), indicating that the aromatic reactive sites remaining after coagulation and softening had a higher potential for THM formation than those in the raw water. Including all data points (unfractionated and SPE filtrates of both raw and treated water) resulted in a moderate correlation between SUVA and STHMFP (Figure 5b; *R* ^2^ = 0.4636).

The circled data of HPI fractions in Figure 5 demonstrates the higher STHMFP of HPI DOC in both raw and treated water. This visualization also revealed no clear correlation between SUVA and STHMFP. Other studies also did not observe a robust correlation between SUVA and THMFP (Weishaar et al., 2003). In both unfractionated samples and SPE filtrates, the Soft/Filt samples exhibited higher STHMFP despite having lower SUVA values than the raw fractions. However, it is worth noting that lower SUVA values may be associated with lower DOC concentrations and, consequently, lower total THMFP, particularly for unfractionated samples where UV absorbance strongly correlates with DOC. The discussion suggests that STHMFP, like SUVA, does not appear to be inherently linked to whether the DOC is HPO or HPI. Instead, the reactivity of DOC with chlorine is likely influenced by the specific interactions and chemical characteristics of the organic compounds present in the water.

### Proposed DOC fractionation method using two ENV cartridges

**FIGURE 6:**
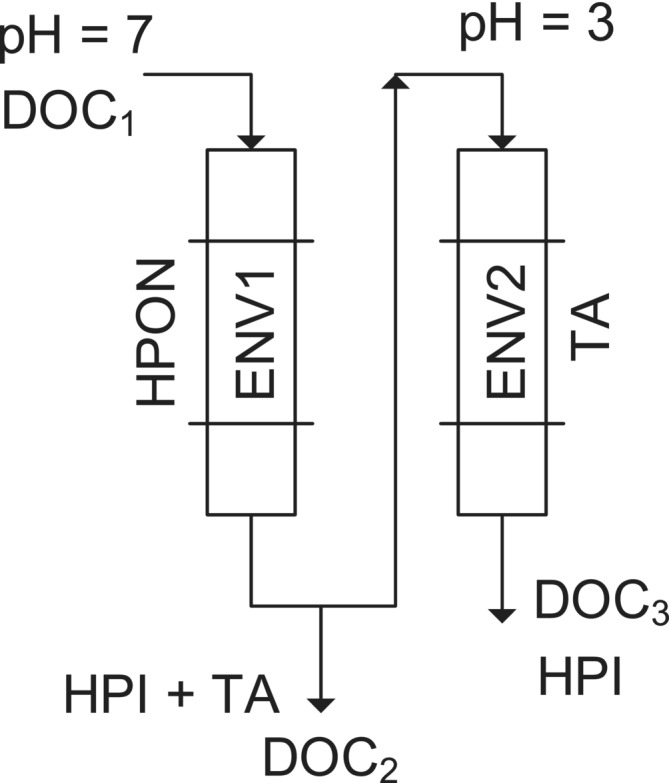
Proposed fractionation method to estimate HPON, TA, and HPI DOC using two ENV cartridges. HPI DOC, hydrophilic dissolved organic carbon; HPON, hydrophobic neutral; TA, total acidic.

The existing fractionation methods require recovering the adsorbed DOC from the resin, which can be challenging. Therefore, we propose a new fractionation method to estimate major DOC fractions, including HPON, TA, and HPI without the need to recover the DOC from the resin. This new method employs a Bond Elut ENV cartridge at pH 7 to retain the HPON DOC, followed by a second ENV extraction at pH 3 to isolate the TA DOC (Figure 6), while the HPI DOC remains in the final effluent. At pH 7, the DOC retained by the ENV cartridge represents the HPO fraction at neutral pH, the HPON. At pH 3, the retained compounds are those that become HPO as the pH decreases (Newcomb et al., 2017), that is, TA fraction. The DOC that remains in the SPE filtrate at pH 3 is the intrinsic HPI DOC, consisting of organic compounds that remain HPI even at low pH levels. This sequential approach provides an efficient way to estimate the key DOC fractions without the complications associated with direct recovery of the adsorbed compounds. The DOC and UV should be measured on unfractionated water sample, the effluent of ENV1 and ENV2 to calculate the content and mass fraction ($x_{i}$) of DOC fractions using Equations (1)–(3).

(1) $$\mathrm{HPI}\;\mathrm{DOC}={\mathrm{DOC}}_{3};x_{\mathrm{HPI}}=\mathrm{HPI}\;\mathrm{DOC}/{\mathrm{DOC}}_{1}\;$$

(2) $$\text{HPON}\;\mathrm{DOC}={\mathrm{DOC}}_{1}-{\mathrm{DOC}}_{2};x_{\text{HPON}}=\text{HPON}\;\mathrm{DOC}/{\mathrm{DOC}}_{1}\;$$

(3) $$\mathrm{TA}\;\mathrm{DOC}={\mathrm{DOC}}_{2}-{\mathrm{DOC}}_{3};x_{\mathrm{TA}}=\mathrm{TA}\;\mathrm{DOC}/{\mathrm{DOC}}_{1}\;$$

Because SUVA is an intensive parameter, the SUVA of a water sample reflects the combined contributions of its various components and can be expressed as $\text{SUVA}=\sum x_{i}\times {\text{SUVA}}_{i}$, where $x_{i}$ is the mass fraction of the component i (Kitis et al., 2002). Therefore, the SUVA of major DOC fractions can be estimated approximately using Equations (4)–(6). The contribution of each fraction to the aromaticity of the Raw and Soft/Filt samples was calculated by dividing the SUVA of each fraction by the SUVA of the unfractionated sample. The results of the new fractionation method, as shown in Table 5, revealed notable changes in both the composition and characteristics of DOC fractions between the raw and softened/filtered water samples.

(4) $${\text{SUVA}}_{\mathrm{HPI}}={\text{SUVA}}_{3}$$

(5) $${\text{SUVA}}_{\text{HPON}}=\left[{\text{SUVA}}_{1}-\left(x_{2}\times {\text{SUVA}}_{2}\right)\right]/x_{\text{HPON}}\;$$

(6) $${\text{SUVA}}_{\mathrm{TA}}=\begin{array}{l} {}[{\text{SUVA}}_{1}-\left(x_{3}\times {\text{SUVA}}_{3}\right)\\ -\;\left(x_{\text{HPON}}\times {\text{SUVA}}_{\text{HPON}}\right)]/x_{\mathrm{TA}} \end{array}$$

**TABLE 5:** The quantity and SUVA of DOC fractions isolated using ENV_pH = 3_ and ENV_pH = 7_.

DOC fractions		DOC (mg/l)	Fraction (%)	SUVA (l/mg‐C·m)	Contribution to aromaticity (%)
Raw					
	HPI	1.14	14	15.1	86
	HPON	4.31	53	0	0
	TA	2.63	33	1.33	17
**Soft/Filt**					
	HPI	1.19	25	6.3	89
	HPON	1.35	29	0.96	15
	TA	2.20	46	0	0

*Note*: DOC, dissolved organic carbon; HPI, hydrophilic; HPON, hydrophobic neutral; TA, total acidic; SUVA, specific UV_254_ absorption.

The raw water was composed of 14% HPI, 53% HPON, and 33% TA fractions. After treatment, the distribution shifted, with the HPI fraction increasing to 25%, TA rising to 46%, and HPON decreasing to 29%. This change suggests that the treatment process significantly reduced HPON compounds while increasing the relative amounts of HPI and TA fractions. In raw water, the HPI fraction exhibited a high SUVA of 15.1 l/mg‐C·m, contributing 86% of the total aromaticity. The HPON fraction, which made up the largest portion of DOC in raw water at 53%, had a SUVA of 0.0 l/mg‐C·m, indicating minimal aromaticity. The TA fraction had a SUVA of 1.3 l/mg‐C·m, contributing 17.4% to the total aromaticity. After treatment, the SUVA of the HPI fraction decreased to 6.3 l/mg‐C·m, reflecting a reduction in aromaticity, while its contribution to total aromaticity remained similar. The HPON fraction decreased to 29% of the DOC, with its SUVA slightly increasing to 1.0 l/mg‐C·m, while the SUVA of the TA fraction dropped to 0.0 l/mg‐C·m, indicating changes in the aromatic content of these fractions. Despite these shifts, the contributions of HPON and TA to the total aromaticity remained low after treatment.

Most studies did not consider the changes in DOC fraction characteristics, assuming that treatment processes affect only the quantity of specific DOC fractions rather than their properties. As a result, researchers have often characterized DOC fractions using SUVA, THMFP, functional groups, and structural carbon distribution solely in raw water and not in treated water (Bose & Reckhow, 1998; J.‐P. P. Croué et al., 2000; Goss & Gorczyca, 2013; Ratpukdi et al., 2010; Sadrnourmohamadi et al., 2013). However, 1H nuclear magnetic resonance (NMR) analysis of resin‐fractionated HPO has shown that the HPO fraction in water treated with polyaluminum chloride (PACl) contains more aromatics and fewer aliphatic hydrogen bonds (H. C. Kim & Yu, 2005). Another recent study demonstrated that although HPON and HPOA fractions isolated by sequential SPE had fewer carbon double bonds and more C–O bonds post‐ozonation, they still remained within their initial HPON and HPOA categories (Phungsai et al., 2021).

Although SUVA is a reliable indicator for relative abundance of aromatic and highly conjugated organic compounds within NOM and treatability of DOC by conventional or enhanced coagulation processes, its application to determine hydrophobicity of DOC has shown inconsistencies. For example, Bose and Reckhow found that samples varying levels of hydrophobicity in DOC (56 to 79%) had similar SUVA values (Bose & Reckhow, 1998). Additionally, the HPOA and HPIN fractions exhibited comparable SUVA values, 4.3 versus 3.9 l/mg‐C m (Bose & Reckhow, 1998). Our data suggest that aromaticity determined by SUVA may not be specific to either HPO or HPI fractions and vary after treatment units. The HPI DOC can exhibit significant UV_254_ absorbance depending on the chemical structure of the original compounds and the applied treatment processes.

## CONCLUSIONS

We investigated the effects of various fractionation methods on the assessment of major DOC fractions in water treatment processes. Most existing methods focus on isolating HPO fractions by eluting them from the resin, which complicates cross‐study comparisons because of varying recovery rates. Additionally, many studies did not consider changes in DOC fraction characteristics during treatment, assuming that unit processes only affect the quantity of DOC fractions, not their properties. To address this, we proposed a fractionation method that estimates the quantity and characteristics of major DOC fractions—HPIN, TA (TA = HPOA + HPIA), and HPON—without the need to recover sorbed fractions. Three pH approaches (pH 3, pH 7, and sequential pH adjustment) and three SPE types (ENV, C18, and C18‐EWP) were employed. The following conclusions can be drawn from this study:

- Based on a thorough literature review, the major DOC fractions were HPI, TA, and HPON. We estimated these fractions using a new fractionation method, employing one ENV cartridge at pH 3 and another at pH 7, without eluting the sorbed DOC from the resins.
- The new fractionation method revealed that conventional coagulation/softening significantly altered the content of DOC fractions. While the HPO neutral compounds decreased, the relative proportions of HPI and TA fractions increased, shifting from 15% to 28% and 33% to 54%, respectively, highlighting changes in DOC composition in the treatment process.
- The study showed that the conventional softening significantly reduced the SUVA of the HPI fraction. However, HPI contribution to total SUVA increased because of the substantial drop in the TA fraction's SUVA. These results suggest that SUVA should not be used as an indicator of water HPO or HPI composition.
- The HPI DOC fractions in Raw and Soft/Filt water samples exhibited significantly higher SUVA values, up to six times, compared to their unfractionated counterparts. This increase was because of minimal changes in UV_254_ absorbance and a reduction in DOC concentrations. The high SUVA for HPI DOC components is because of their chemical structure and intramolecular interactions.
- The HPI DOC in the ENV_pH = 3_ filtrate represented the inherently HPI fraction of DOC and compared well in quantity, MW size, SUVA, and THMFP with the HPI obtained using the sequential ENV. Hence, one ENV isolation at pH = 3 can replace three ENV cartridges for isolating the HPI DOC.

The data and conclusions suggest that UV_254_ absorbance, MW size, and STHMFP are not related to DOC hydrophobicity but rather depend on the chemical characteristics and interactions of the organic compounds present. Isolating the DOC fraction using ENV cartridges at pH 3 and 7 can effectively quantify and characterize intrinsic HPI DOC, acidic‐ionizable DOC, and HPON DOC. This fractionation method does not require eluting any compounds from the resin and may allow for better comparison between the different studies.

## AUTHOR CONTRIBUTIONS

**Saeideh Mirzaei:** Conceptualization; methodology; investigation; formal analysis; visualization; project administration; writing – original draft. **Beata Gorczyca:** Supervision; funding acquisition; conceptualization; methodology; writing – review and editing.

## CONFLICT OF INTEREST STATEMENT

The authors declare no conflict of interest.

## Supporting information


**Figure S1.** Brandon WTP and the sampling points: Raw, Soft W, and Soft/Filt W
**Figure S2.** Correlation between DOC and UV_254_ of HA Std. Including unfractionated samples and HPI fractions. The two points on the right end of the figure belong to a new HA stock solution with DOC of 7.563 and its associated HPI fraction at pH = 7.
**Table S1.** SUVA Guideline for assessing the nature and removal of DOC in (advanced) coagulation (Archer & Singer, 2006; Edzwald & Tobiason, 1999).
**Table S2.** Content of various DOC fractions isolated using resin fractionation or SPE cartridges. A dash (−) indicates data not provided in the reviewed study; ‘n.a.’ means not applicable for the fractionation method used. Gray rows represent different water samples within the same study.
**Table S3.** Properties of applied solid‐phase extraction cartridges from Bond Elut family based on the manufacturer (Agilent) information
**Table S4.** water quality parameters at Brandon WTP, presented as the mean ± one standard deviation from three sampling dates.

## Data Availability

The data that support the findings of this study are openly available in Mendeley Data at https://data.mendeley.com/datasets/dbs9nps58j/3, reference number 10.17632/dbs9nps58j.3.
