# Supplementary material for: Quantifying and characterizing major DOC fractions in water treatment processes: A simplified SPE method without recovering sorbed compounds
Source: Water Environ Res. 2025 Feb 20;97(2):e70047. doi: 10.1002/wer.70047 (PMC11840460; doi:10.1002/wer.70047)
Supplement: Supplementary file 1 — Figure S1. Brandon WTP and the sampling points: Raw, Soft W, and Soft/Filt W Figure S2. Correlation between DOC and UV254 of HA Std. Including unfractionated samples and HPI fractions. The two points on the right end of the figure belong to a new HA stock solution with DOC of 7.563 and its associated HPI fraction at pH = 7. Table S1. SUVA Guideline for assessing the nature and removal of DOC in (advanced) coagulation (Archer & Singer, 2006; Edzwald & Tobiason, 1999). Table S2. Content of various DOC fractions isolated using resin fractionation or SPE cartridges. A dash (−) indicates data not provided in the reviewed study; ‘n.a.’ means not applicable for the fractionation method used. Gray rows represent different water samples within the same study. Table S3. Properties of applied solid‐phase extraction cartridges from Bond Elut family based on the manufacturer (Agilent) information Table S4. water quality parameters at Brandon WTP, presented as the mean ± one standard deviation from three sampling dates. [file WER-97-e70047-s001.docx]

**Quantifying and Characterizing Major DOC Fractions in Water Treatment Processes: A Simplified SPE Method Without Recovering Sorbed Compounds**

Saeideh Mirzaei^a^, Beata Gorczyca^a*^

^a^ Department of Civil Engineering, University of Manitoba, 15 Gillson St., E1-368 EITC, Winnipeg, MB R3T 5V6, Canada

*Corresponding author: Tel: +1-204-474-6674, E-mail: Beata.Gorczyca@umanitoba.ca

Figure S1. Brandon WTP and the sampling points: Raw, Soft W, and Soft/Filt W

|  | 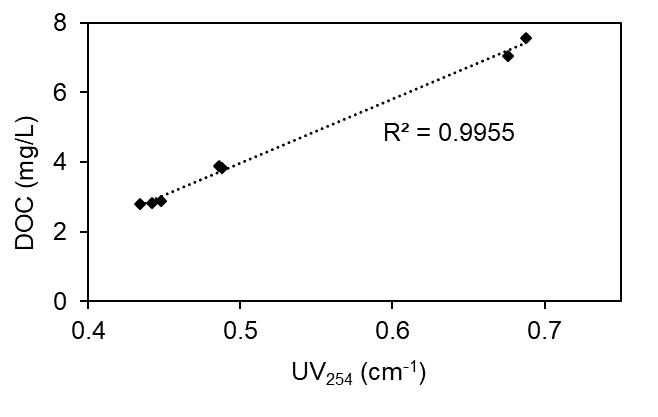 |
| --- | --- |

Figure S2. Correlation between DOC and UV_254_ of HA Std. Including unfractionated samples and HPI fractions. The two points on the right end of the figure belong to a new HA stock solution with DOC of 7.563 and its associated HPI fraction at pH=7.

Table S1. SUVA Guideline for assessing the nature and removal of DOC in (advanced) coagulation (Archer & Singer, 2006; Edzwald & Tobiason, 1999).

| **SUVA (L/mg-C m)** | **DOC Composition** | **DOC removal** |
| --- | --- | --- |
| 1-2 | Low hydrophobicity, low MW | 25-35% |
| 2-4 | A mixture of HPO and HPI organics, a mixture of MWs | ~ 40% |
| > 4 | High hydrophobicity, high MW | >50% |

Table S2. Content of various DOC fractions isolated using resin fractionation or SPE cartridges. A dash (-) indicates data not provided in the reviewed study; 'n.a.' means not applicable for the fractionation method used. Gray rows represent different water samples within the same study.

| Water sample | | | Water characteristics | | Fractionation method | | HPO (%)  [Recovery (%)] | | | HPI (%)  [Recovery (%)] | | | TP**^1^** | Missing DOC (%) | Analyses on DOC fractions | Reference |
| --- | --- | --- | --- | --- | --- | --- | --- | --- | --- | --- | --- | --- | --- | --- | --- | --- |
|  |  |  | DOC (mg/L) | SUVA (L/mg-C m) |  |  | HPON  (%) | HPOA  (%) | HPOB  (%) | HPIB  (%) | HPIA  (%) | HPIN  (%) |  |  |  |  |
| South Platte River | | | 7.5 | - | XAD-8, Bio Rad AG-MP-50, Duolite A-7 | | 51 | | | 49 | | | n.a. |  | Functional groups by FTIR | (Leenheer, 1981) |
|  |  |  |  |  |  |  | 31.6  [130] | 18.0  [100] | 1.2  [100] | 25.4  [102] | 18.1  [126] | 5.6  [n.a.] |  |  |  |  |
| Raw water | Everette WTP | | 1.7 | - | XAD-8, Bio Rad AG-MP-50, Duolite A-7 | | 55 | | | 46 | | | n.a. | - | Carbon-type distribution by NMR, TOXFP**^2^**, UV_254_ | (Korshin, Benjamin, & Sletten, 1997) |
|  |  |  |  |  |  |  | 0 | 53 | 2 | 1 | 37 | 8 |  |  |  |  |
|  | Vernon WTP | | 4.6 | - |  |  | 52 | | | 48 | | |  | - |  |  |
|  |  |  |  |  |  |  | 8 | 43 | 1 | 3 | 40 | 5 |  |  |  |  |
| Raritan/Millstone WTP, USA | Raw W | | 4.0 | - | XAD-8, Bio Rad AG-MP-50, Duolite A-7 | | 36.4 | | | 73.0 | | | n.a. | -9.35**^3^** | - | (Marhaba, Van, & Lippincott, 2000) |
|  |  |  |  |  |  |  | 18.3 | 12.1 | 6.0 | 3.9 | 48.3 | 20.8 |  |  |  |  |
|  | Coagulation/  sedimentation | | 3.0 | - |  |  | 39.5 | | | 73.6 | | |  | -13.1 |  |  |
|  |  |  |  |  |  |  | 21.9 | 11.6 | 6.0 | 7.2 | 56.3 | 10.1 |  |  |  |  |
|  | Filtration | | 2.0 | - |  |  | 35.5 | | | 72.5 | | |  | -8.04 |  |  |
|  |  |  |  |  |  |  | 21.4 | 8.8 | 5.3 | 6.1 | 60.0 | 6.4 |  |  |  |  |
|  | Clearwell | | 2.0 | - |  |  | 36.0 | | | 66.6 | | |  | -2.53 |  |  |
|  |  |  |  |  |  |  | 22.1 | 8.5 | 5.4 | 7.0 | 50.5 | 9.1 |  |  |  |  |
| Canal Road WTP, NJ, USA | Raw | | 4.0 | - | XAD-8, Bio Rad AG-MP-50, Duolite A-7 | | 36.4 | | | 73.0 | | | n.a. | -9.35 | - | (Marhaba et al., 2000) |
|  |  |  |  |  |  |  | 18.3 | 12.1 | 6.0 | 3.9 | 48.3 | 20.8 |  |  |  |  |
|  | Ozonation | | 4.0 | - |  |  | 28.1 | | | 78.8 | | |  | -6.92 |  |  |
|  |  |  |  |  |  |  | 18.2 | 9.5 | 0.4 | 6.6 | 51.3 | 20.9 |  |  |  |  |
|  | Coagulation/  sedimentation | | 3.0 | - |  |  | 20.9 | | | 79.4 | | |  | -0.34 |  |  |
|  |  |  |  |  |  |  | 12.7 | 7.9 | 0.4 | 4.6 | 51.5 | 23.3 |  |  |  |  |
|  | Filtration | | 2.0 | - |  |  | 21.9 | | | 89.3 | | |  | -11.14 |  |  |
|  |  |  |  |  |  |  | 15.1 | 6.3 | 0.4 | 5.5 | 57.0 | 26.7 |  |  |  |  |
|  | Clearwell | | 2.0 | - |  |  | 23.4 | | | 87.3 | | |  | -10.66 |  |  |
|  |  |  |  |  |  |  | 16.7 | 6.3 | 0.4 | 4.9 | 54.2 | 28.2 |  |  |  |  |
| Surface waters | Myrtle Beach | | 20.2 | 4.65 | XAD-8 at pH=2 | | 66  [105.6] | | | 34  [n.a] | | | n.a. | - | UV_254_, THMFP, HHAFP | (Kitis, Karanfil, Wigton, & Kilduff, 2002) |
|  | Tomhannock | | 3.3 | 2.09 |  |  | 36  [89.9] | | | 64  [n.a.] | | |  |  |  |  |
| Gueui WTP, Korea | Raw water | | 2.06 | 1.8 | XAD-7 HP at pH=2 | | 47.2 | | | 52.8 | | | n.a. | - | Carbon-type distribution by NMR, functional groups by FTIR, THMFP, HHAFP | (Kim & Yu, 2005) |
|  | Pre-chlorination | | 2.0 | 1.5 |  |  | 45.5 | | | 53.2 | | |  |  |  |  |
|  | Coagulation/ sedimentation | | 1.9 | 0.7 |  |  | 39.2 | | | 55.3 | | |  |  |  |  |
|  | Filtration | | 1.53 | 0.9 |  |  | 26.4 | | | 48.2 | | |  |  |  |  |
| Concentrated NOM from the Nakdong River | | | 2.25 | 1.81 | 3.5 kDa Dialysis, XAD-8, XAD-4 | | 40.9 | | | 26.9 | | | 19.8 | 12.2 | UV_254_, MW, %BDOC/DOC, HAAFP **^4^** | (Kwon et al., 2005) |
| Forge Pond | | | 3.45 | - | XAD-8, XAD-4, Duolite A-7 | | 57.9 | | | 33.1 | | | n.a. | 8.33 | Organic charge at pH =7 , UV_254_ | (Bose & Reckhow, 2007) |
|  |  |  |  |  |  |  | 7.9 | 48.4 | 1.6 | 2.8 | 8.1 | 22.2 |  |  |  |  |
| WTPs’ intakes at | | Repentigny | 7.1 | 4.4 | XAD-8, XAD-4 | | 56.3 | | | 19.7 | | | 22.5 | - | UV_254_, THMFP and HAAFP, , TOXFP, Br^-^ | (Hua & Reckhow, 2007) |
|  |  | Waco | 4.0 | 2.8 |  |  | 52.5 | | | 27.5 | | | 27.5 |  |  |  |
|  |  | Winnipeg | 7.9 | 1.6 |  |  | 43.0 | | | 26.6 | | | 27.8 |  |  |  |
|  |  | Springfield | 3.3 | 3.5 |  |  | 64.0 | | | 36.0 | | | - |  |  |  |
|  |  | Tampa | 12.9 | 4.4 |  |  | 67.0 | | | 33.0 | | | - |  |  |  |
| Source water of Tiangin’s WTP | | | 3.78 | 2.14 | XAD-8, Bio Rad AG-MP-50, Duolite A-7 | | 51.5 | | | 48.5 | | | n.a. | - | THMFP and HAAFP | (Chen et al., 2008) |
|  |  |  |  |  |  |  | 12.3 | 35.6 | 3.6 | 4.1 | 26.5 | 17.9 |  |  |  |  |
| Suwannee River | | | 6.22 | 3.84 | Sequential SPE | | 73.4 | | | 26.4 | | | n.a. | - | Carbon-type distribution by NMR, functional groups by FTIR | (Ratpukdi, Rice, Chilom, Bezbaruah, & Khan, 2009) |
|  |  |  |  |  |  |  | 2.55  [75] | 69.8  [79] | 1.08  [2] | 3.27  [102] | 14.66  [64] | 8.46  [n.a.] |  |  |  |  |
| Moorhead WTP, USA | Raw water  (Red River) | | 8.44 | 2.41 | Sequential SPE | | 53.7 | | | 46.3 | | | n.a. | - | Carbon-type distribution by NMR, functional groups by FTIR | (Ratpukdi et al., 2009) |
|  |  |  |  |  |  |  | 15.68  [96] | 35.43  [101] | 2.59  [103] | 3.44  [78] | 17.95  [97] | 24.91  [n.a.] |  |  |  |  |
|  | Coagulation/  softening | | 4.22 | 1.73 |  |  | 52.4 | | | 52.1 | | | n.a. | - | none |  |
|  |  |  |  |  |  |  | 16.59 | 33.18 | 2.61 | 3.55 | 10.66 | 37.86 |  |  |  |  |
|  | Ozonation | | 3.71 | 0.78 |  |  | 30.2 | | | 73.3 | | | n.a. | - | none |  |
|  |  |  |  |  |  |  | 5.28 | 24.91 | 0 | 0 | 18.19 | 55.13 |  |  |  |  |
|  | Filtration | | 2.97 | 0.88 |  |  | 33.5 | | | 69.7 | | | n.a. | - | none |  |
|  |  |  |  |  |  |  | 6.60 | 26.94 | 0 | 0 | 23.57 | 46.14 |  |  |  |  |
| Coagulated/soften-ed Red River, Moorhead WTP, USA | | | 4.18 | 1.04 | Sequential SPE | | 50.8 | | | 49.5 | | | n.a. | - | none | (Ratpukdi, Siripattanakul, & Khan, 2010) |
|  |  |  |  |  |  |  | 14.8 | 34.8 | 1.0 | 3.9 | 7.3 | 38.3 |  |  |  |  |
| River Oulujoki**^5^** | | | 7.9-14.5 | - | XAD-8, XAD-4 | | 63-67 | | | 15-19 | | | 15-20 | - | none | (Mikola, Rämö, Sarpola, & Tanskanen, 2013) |
| Red River | | | 11.78 | 2.36 | Sequential SPE | | 36.8 | | | 63.3 | | | n.a. | - | none | (Sadrnourmohamadi, Goss, & Gorczyca, 2013) |
|  |  |  |  |  |  |  | 12.1 | 23.3 | 1.4 | 11.2 | 7.9 | 44.23 |  |  |  |  |
| Red River | Sep. 2010 | | 11.3 | - | Sequential SPE | | 46.0 | | | 55.0 | | | n.a. | - | none | (Goss & Gorczyca, 2013) |
|  |  |  |  |  |  |  | 22 | 22 | 2 | 2 | 13 | 40 |  |  |  |  |
|  | Nov. 2010 | | 12.0 | 2.9 |  |  | 57.7 | | | 42.2 | | |  |  |  |  |
|  |  |  |  |  |  |  | 18.2 | 35.9 | 3.6 | 5.5 | 11.6 | 25.1 |  |  |  |  |
|  | Feb. 2011 | | 8.02 | 3.4 |  |  | 51.6 | | | 37.4 | | |  |  |  |  |
|  |  |  |  |  |  |  | 11.7 | 33.1 | 6.8 | 6.2 | 15.2 | 16 |  |  |  |  |
|  | Jun. 2011 | | 8.65 | - |  |  | 41.0 | | | 59.0 | | |  |  |  |  |
|  |  |  |  |  |  |  | 11.5 | 27.1 | 2.4 | 5.3 | 2.3 | 51.4 |  |  |  |  |
|  | Aug. 2011 | | 9.8 | - |  |  | 47.0 | | | 53.6 | | |  | - | THMFP |  |
|  |  |  |  |  |  |  | 13 | 32.5 | 1.5 | 6 | 5.6 | 42 |  |  |  |  |
| Portage la Prairie WTP supplied by Assiniboine River | Raw Water | | 16.2 | 2.92**^6^** | Sequential SPE | | 38.4 | | | 61.6 | | | n.a. | - | THMFP | (Goss & Gorczyca, 2013) |
|  |  |  |  |  |  |  | 5.8 | 31.4 | 1.2 | 4.9 | 15.3 | 41.3 |  |  |  |  |
|  | Actiflo Coagulation | | 17.5 | - |  |  | 52.4 | | | 47.6 | | |  | - | none |  |
|  |  |  |  |  |  |  | 15.5 | 31.4 | 5.5 | 4.5 | 13.3 | 29.8 |  |  |  |  |
|  | Softening | | 11.1 | - |  |  | 48.5 | | | 51.5 | | |  |  |  |  |
|  |  |  |  |  |  |  | 13.6 | 34.2 | 0.7 | 4.9 | 12.0 | 34.6 |  |  |  |  |
|  | Carbonation | | 7.0 | - |  |  | 44.9 | | | 55.1 | | |  |  |  |  |
|  |  |  |  |  |  |  | 10.5 | 32.6 | 1.8 | 3.6 | 9.1 | 42.5 |  |  |  |  |
|  | Ozonation | | 9.5 | - |  |  | 44.4 | | | 55.6 | | |  |  |  |  |
|  |  |  |  |  |  |  | 9.0 | 32.9 | 2.5 | 0.2 | 13.4 | 42.0 |  |  |  |  |
|  | Sand filter  reservoir | | 7.9 | - |  |  | 28.1 | | | 60.2 | | |  |  |  |  |
|  |  |  |  |  |  |  | 5.3 | 20.9 | 1.9 | 3.2 | 7.0 | 49.9 |  |  |  |  |
|  | GAC filter | | 7.2 | - |  |  | 31.1 | | | 70.2 | | |  |  |  |  |
|  |  |  |  |  |  |  | 2.1 | 29.0 | 0.0 | 7.7 | 14.1 | 48.5 |  |  |  |  |
|  | Clear well | | 9.1 | - |  |  | 50.8 | | | 76.4 | | |  |  |  |  |
|  |  |  |  |  |  |  | 6.4 | 39.4 | 5.1 | 4.2 | 3.0 | 69.2 |  |  |  |  |
| Seven water sources in Eastern North America | Pond | | 3.6 | 3.6 | XAD-8, Bio Rad AG-MP-50, Duolite A-7 | | 36 | | | 65 | | | n.a. | - | Size distribution using UV-HPSEC, UV_254_ | (Kent, Montreuil, Stoddart, Reed, & Gagnon, 2014) |
|  |  |  |  |  |  |  | 3 | 30 | 3 | 2 | 9 | 54 |  |  |  |  |
|  | Pond | | 7.3 | 6.2 |  |  | 26 | | | 74 | | |  |  |  |  |
|  |  |  |  |  |  |  | 3 | 22 | 1 | 2 | 51 | 21 |  |  |  |  |
|  | River | | 5.3 | 1.8 |  |  | 41 | | | 57 | | |  |  |  |  |
|  |  |  |  |  |  |  | 4 | 35 | 2 | 1 | 6 | 50 |  |  |  |  |
|  | Lake | | 5.6 | n.a. |  |  | 48 | | | 52 | | |  |  |  |  |
|  |  |  |  |  |  |  | 4 | 40 | 4 | 1 | 13 | 38 |  |  |  |  |
|  | Lake | | 3.1 | 1.9 |  |  | 34 | | | 66 | | |  |  |  |  |
|  |  |  |  |  |  |  | 1 | 28 | 5 | 1 | 12 | 53 |  |  |  |  |
|  | River | | 8.3 | 5.1 |  |  | 62 | | | 38 | | |  |  |  |  |
|  |  |  |  |  |  |  | 3 | 58 | 1 | 1 | 3 | 34 |  |  |  |  |
|  | Pond | | 9.0 | 4.5 |  |  | 72 | | | 28 | | |  |  |  |  |
|  |  |  |  |  |  |  | 2 | 63 | 7 | 1 | 12 | 15 |  |  |  |  |
| Lake Inbanuma | | | - | 2.1 | XAD-8, XAD-4 | | 49 | | | 23 | | | 28 | - | UV_260_, carbohydrate content, fluorescence EEMs, functional groups by FTIR | (Yamamura, Okimoto, Kimura, & Watanabe, 2014) |
| Kushiro River | | | - | 4.2 |  |  | 50 | | | 20 | | | 30 |  |  |  |
| Yodo River | | | - | 2.0 |  |  | 53 | | | 31 | | | 16 |  |  |  |
| Toyohira River | | | 0.8 | 2.8**^7^** |  |  | 60 | | | 20 | | | 20 |  |  |  |
| Assiniboine River | | | 13.8 | 3.4 | Sequential SPE | | 71 | | | 29 | | | n.a. | - | none | (Sadrnourmohamadi & Gorczyca, 2015) |
|  |  |  |  |  |  |  | 19.6 | 50.1 | 1.3 | 3.33 | 6.45 | 19.2 |  |  |  |  |
| Coagulated/filtered water | Autumn | | 0.84 | - | DAX-8,  XAD-4 | | 40.1 | | | 42.0 | | | 17.9 | - | UV_254_, Br-, chlorine demand, NH_4_^+^ | (Phattarapattamawong, Echigo, & Itoh, 2016) |
|  |  |  |  |  |  |  | 7.2 | 32.3 | 0.6 | 9.6 | 22.8 | 9.6 |  |  |  |  |
|  | Spring | | 0.86 | - |  |  | 37.3 | | | 44.3 | | | 18.6 |  |  |  |
|  |  |  |  |  |  |  | 8.7 | 28.6 | n.g. | 11.7 | 15.6 | 17 |  |  |  |  |
| La Salle River | | | 18.3 | - | Different types of SPE cartridges at pH = 2 | ENV | 46.6 | | | 53.4 | | | n.a. | 11.6**^8^** | THMFP, functional groups by FTIR | (Goss, Wiens, Gorczyca, & Gough, 2017) |
|  |  |  |  |  |  | PPL | 50.2 | | | 49.8 | | |  | 9.2 |  |  |
|  |  |  |  |  |  | Strata-X | 46.4 | | | 53.6 | | |  | 15.5 |  |  |
| Lake Winnipegosis | | | 15.8 | - |  | ENV | 36.2 | | | 63.8 | | |  | 13.4 |  |  |
|  |  |  |  |  |  | PPL | 47.9 | | | 52.1 | | |  | 13.1 |  |  |
|  |  |  |  |  |  | Strata-X | 51.6 | | | 48.5 | | |  | 16.8 |  |  |
| Waterhen River | | | 11.4 | - |  | ENV | 28.6 | | | 71.5 | | |  | 14.0 |  |  |
|  |  |  |  |  |  | PPL | 37.3 | | | 62.7 | | |  | 11.6 |  |  |
|  |  |  |  |  |  | Strata-X | 31.9 | | | 68.1 | | |  | 13.9 |  |  |
| Lake Tai, China | | | 3.8 | 2.07 | DAX-8,  XAD-4,  IRA-958 | | 43.5 | | | n.a. | 7.6**^9^** | 41.1 | 7.8 | - | none | (Huang, Lv, Zhou, Hu, & Dong, 2019) |
| Surface waters | A | | 11.4 | 5.0 | DAX-8,  XAD-4 | | 62 | | | 13 | | | 25 | - | none | (Finkbeiner, Moore, Pereira, Jefferson, & Jarvis, 2020) |
|  | B | | 7.6 | 4.3 |  |  | 51 | | | 21 | | | 28 |  |  |  |
|  | C | | 8.4 | 5.6 |  |  | 52 | | | 18 | | | 30 |  |  |  |
| WTP supplied by Ara River, Japan | Raw water  (Ara River) | | 1.09 | 3.76 | PPL_pH=3_ | | 43 | | | 57 | | | n.a. | - | The Molecular formula (C, H, O) was identified on all DOC fractions obtained through sequential SPE but only on HPO fraction retained by PPL resin from water samples across the WTP. | (Phungsai, Kurisu, Kasuga, & Furumai, 2021) |
|  |  |  |  |  | Sequential SPE | | 39 | | | 36 | | |  | 25 |  |  |
|  |  |  |  |  |  |  | 19 | 20 | 0 | 9 | 5 | 22 |  |  |  |  |
|  | Coagulation/  sedimentation | | 0.96 | 2.71 | PPL_pH=3_ | | 49 | | | 51 | | | n.a. | - |  |  |
|  |  |  |  |  | Sequential SPE | | 38 | | | 44 | | |  | 18 |  |  |
|  |  |  |  |  |  |  | 21 | 17 | 0 | 9 | 6 | 29 |  |  |  |  |
|  | Ozonation | | 0.9 | 1.89 | PPL_pH=3_ | | 42 | | | 58 | | | n.a. | - |  |  |
|  |  |  |  |  | Sequential SPE | | 25 | | | 67 | | |  | 8 |  |  |
|  |  |  |  |  |  |  | 16 | 9 | 0 | 14 | 11 | 42 |  |  |  |  |
|  | BAC filteration | | 0.48 | 2.90 | PPL_pH=3_ | | 42 | | | 58 | | | n.a. | - |  |  |
|  |  |  |  |  | Sequential SPE | | 26 | | | 50 | | |  | 24 |  |  |
|  |  |  |  |  |  |  | 11 | 15 | 0 | 17 | 5 | 28 |  |  |  |  |
|  | Chlorination | | 0.47 | 2.54 | PPL_pH=3_ | | 45 | | | 55 | | | n.a. | - |  |  |
|  |  |  |  |  | Sequential SPE | | 29 | | | 47 | | |  | 24 |  |  |
|  |  |  |  |  |  |  | 20 | 9 | 0 | 8 | 3 | 36 |  |  |  |  |

**^1^** Transphilic DOC fraction that was islated in some fractionation methods.

**^2^** Total organo-halide formation potential.

**^3^** Negative values in this column are the percentages of surplus DOC.

**^4^** BDOC and HAAFP stand for biodegradable DOC and haloacetic acid formation potential.

**^5^** The data in this study is a range across different seasons.

**^6^** This value is for another sampling date adapted from (Goss, 2011).

**^7^** UV absorbance was measured at the wavelength of 260 nm.

**^8^** Missing DOC was calculated by deducting the reported HPO recovey from 100.

**^9^** It is the percentage of charged-HPI in this study that can include both basic and acidic HPI.

Table S3. Properties of applied solid-phase extraction cartridges from Bond Elut family based on the manufacturer (Agilent) information

| **SPE type** | **Sorbent type** | **Pore size (Å)** | **Retention properties** |
| --- | --- | --- | --- |
| C18 | Octadecyl bonded phase, silica-based | 60 | The most hydrophobic sorbent, extreme retention of nonpolar compounds |
| C18-EWP |  | 500 | Similar to C18 but with extra-wide pores to allow more efficient extraction of large molecules (>15,000 MW) |
| ENV | Polystyrene divinyl benzene polymer | 450 | Nonpolar with a similar pore size to C18-EWP that allows fast flow-through applications |

Table S4. water quality parameters at Brandon WTP, presented as the mean ± one standard deviation from three sampling dates.

| **Parameter (Unit)** | **Raw** | **Soft** | **Soft/Filt** |
| --- | --- | --- | --- |
| pH | 7.9 ± 0.2 | - | 8.0 ± 0.4 |
| Alkalinity (mg/L as CaCO_3_) | 284 ± 5 | 89 ± 11 | 86 ± 21 |
| Hardness (mg/L as CaCO_3_) | 450 ± 35 | - | 169 ± 3 |
| DOC (mg/L) | 7.74 ± 0.40 | 4.65 ± 0.37 | 4.65 ± 0.37 |
| UV_254_ (cm^-1^) | 0.213 ± 0.010 | 0.078 ± 0.013 | 0.079 ± 0.008 |
| SUVA (L/mg-C m) | 2.76 ± 0.24 | 1.67 ± 0.16 | 1.67 ± 0.08 |

**References**

Bose, P., & Reckhow, D. A. (2007). The effect of ozonation on natural organic matter removal by alum coagulation. Water Research, 41(7), 1516–1524. https://doi.org/10.1016/j.watres.2006.12.027

Chen, C., Zhang, X. jian, Zhu, L. xia, Liu, J., He, W. jie, & Han, H. da. (2008). Disinfection by-products and their precursors in a water treatment plant in North China: Seasonal changes and fraction analysis. Science of the Total Environment, 397(1–3), 140–147. https://doi.org/10.1016/j.scitotenv.2008.02.032

Finkbeiner, P., Moore, G., Pereira, R., Jefferson, B., & Jarvis, P. (2020). The combined influence of hydrophobicity, charge and molecular weight on natural organic matter removal by ion exchange and coagulation. Chemosphere, 238, 124633. https://doi.org/10.1016/j.chemosphere.2019.124633

Goss, C. D. (2011). Characterization of Natural Organic Matter and Trihalomethane Formation Potential for NOM Fractions Isolated From Two Surface Water Sources in Manitoba By. University of Manitoba.

Goss, C. D., & Gorczyca, B. (2013). Trihalomethane formation potential of DOC fractions isolated from two Canadian Prairie surface water sources. Water Science and Technology: Water Supply, 13(1), 114–122. https://doi.org/10.2166/ws.2012.093

Goss, C. D., Wiens, R., Gorczyca, B., & Gough, K. M. (2017). Comparison of three solid phase extraction sorbents for the isolation of THM precursors from manitoban surface waters. Chemosphere, 168, 917–924. https://doi.org/10.1016/j.chemosphere.2016.10.118

Hua, G., & Reckhow, D. A. (2007). Characterization of disinfection by-product precursors based on hydrophobicity and molecular size. Environmental Science & Technology, 41, 3309–3315.

Huang, W., Lv, W., Zhou, W., Hu, M., & Dong, B. (2019). Investigation of the fouling behaviors correlating to water characteristics during the ultrafiltration with ozone treatment. Science of the Total Environment, 676, 53–61. https://doi.org/10.1016/j.scitotenv.2019.04.271

Kent, F. C., Montreuil, K. R., Stoddart, A. K., Reed, V. A., & Gagnon, G. A. (2014). Combined use of resin fractionation and high performance size exclusion chromatography for characterization of natural organic matter. Journal of Environmental Science and Health, Part A, 49(14), 1615–1622. https://doi.org/10.1080/10934529.2014.950926

Kim, H. C., & Yu, M. J. (2005). Characterization of natural organic matter in conventional water treatment processes for selection of treatment processes focused on DBPs control. Water Research, 39(19), 4779–4789. https://doi.org/10.1016/j.watres.2005.09.021

Kitis, M., Karanfil, T., Wigton, A., & Kilduff, J. E. (2002). Probing reactivity of dissolved organic matter for disinfection by-product formation using XAD-8 resin adsorption and ultrafiltration fractionation. Water Research, 36(15), 3834–3848. https://doi.org/10.1016/S0043-1354(02)00094-5

Korshin, G. V., Benjamin, M. M., & Sletten, R. S. (1997). Adsorption of natural organic matter (NOM) on iron oxide: Effects on NOM composition and formation of organo-halide compounds during chlorination. Water Research, 31(7), 1643–1650. https://doi.org/10.1016/S0043-1354(97)00007-9

Kwon, B., Lee, S., Cho, J., Ahn, H., Lee, D., & Shin, H. S. (2005). Biodegradability, DBP formation, and membrane fouling potential of natural organic matter: Characterization and controllability. Environmental Science and Technology, 39(3), 732–739. https://doi.org/10.1021/es049919z

Leenheer, J. A. (1981). Comprehensive Approach to Preparative Isolation and Fractionation of Dissolved Organic Carbon from Natural Waters and Wastewaters. Environmental Science and Technology, 15(5), 578–587. https://doi.org/10.1021/es00087a010

Marhaba, T. F., Van, D., & Lippincott, R. L. (2000). Changes in NOM fractionation through treatment: A comparison of ozonation and chlorination. Ozone: Science and Engineering, 22(3), 249–266. https://doi.org/10.1080/01919510008547209

Mikola, M., Rämö, J., Sarpola, A., & Tanskanen, J. (2013). Removal of different NOM fractions from surface water with aluminium formate. Separation and Purification Technology, 118, 842–846. https://doi.org/10.1016/j.seppur.2013.08.037

Phattarapattamawong, S., Echigo, S., & Itoh, S. (2016). Characterization of organic precursors for chlorinous odor before and after ozonation by a fractionation technique. Water Research, 88, 836–843. https://doi.org/10.1016/j.watres.2015.11.001

Phungsai, P., Kurisu, F., Kasuga, I., & Furumai, H. (2021). Changes in dissolved organic matter during water treatment by sequential solid-phase extraction and unknown screening analysis. Chemosphere, 263, 128278. https://doi.org/10.1016/j.chemosphere.2020.128278

Ratpukdi, T., Rice, J. A., Chilom, G., Bezbaruah, A., & Khan, E. (2009). Rapid Fractionation of Natural Organic Matter in Water Using a Novel Solid-Phase Extraction Technique. Water Environment Research, 81(11), 2299–2308. https://doi.org/10.2175/106143009X407302

Ratpukdi, T., Siripattanakul, S., & Khan, E. (2010). Mineralization and biodegradability enhancement of natural organic matter by ozone-VUV in comparison with ozone, VUV, ozone-UV, and UV: Effects of pH and ozone dose. Water Research, 44(11), 3531–3543. https://doi.org/10.1016/j.watres.2010.03.034

Sadrnourmohamadi, M., & Gorczyca, B. (2015). Effects of ozone as a stand-alone and coagulation-aid treatment on the reduction of trihalomethanes precursors from high DOC and hardness water. Water Research, 73(February), 171–180. https://doi.org/10.1016/j.watres.2015.01.023

Sadrnourmohamadi, M., Goss, C. D., & Gorczyca, B. (2013). Removal of DOC and its fractions from surface waters of the Canadian Prairie containing high levels of DOC and hardness. Water Science and Technology: Water Supply, 13(3), 864–870. https://doi.org/10.2166/ws.2013.078

Yamamura, H., Okimoto, K., Kimura, K., & Watanabe, Y. (2014). Hydrophilic fraction of natural organic matter causing irreversible fouling of microfiltration and ultrafiltration membranes. Water Research, 54, 123–136. https://doi.org/10.1016/j.watres.2014.01.024
